# Using Micro-CT Derived Bone Microarchitecture to Analyze Bone Stiffness – A Case Study on Osteoporosis Rat Bone

**DOI:** 10.3389/fendo.2015.00080

**Published:** 2015-05-20

**Authors:** Yuchin Wu, Samer Adeeb, Michael R. Doschak

**Affiliations:** ^1^Department of Biomedical Engineering, University of Alberta, Edmonton, AB, Canada; ^2^Department of Civil and Environmental Engineering, University of Alberta, Edmonton, AB, Canada; ^3^Faculty of Pharmacy and Pharmaceutical Sciences, University of Alberta, Edmonton, AB, Canada

**Keywords:** micro-computed tomography, microstructural parameters, finite element analysis, bone stiffness

## Abstract

Micro-computed tomography (Micro-CT) images can be used to quantitatively represent bone geometry through a range of computed attenuation-based parameters. Nonetheless, those parameters remain indirect indices of bone microarchitectural strength and require further computational tools to interpret bone structural stiffness and potential for mechanical failure. Finite element analysis (FEA) can be applied to measure trabecular bone stiffness and potentially predict the location of structural failure in preclinical animal models of osteoporosis, although that procedure from image segmentation of Micro-CT derived bone geometry to FEA is often challenging and computationally expensive, resulting in failure of the model to build. Notably, the selection of resolution and threshold for bone segmentation are key steps that greatly affect computational complexity and validity. In the following study, we evaluated an approach whereby Micro-CT derived grayscale attenuation and segmentation data guided the selection of trabecular bone for analysis by FEA. We further correlated those FEA results to both two- and three-dimensional bone microarchitecture from sham and ovariectomized (OVX) rats (*n* = 10/group). A virtual cylinder of vertebral trabecular bone 40% in length from the caudal side was selected for FEA, because Micro-CT based image analysis indicated the largest differences in microarchitecture between the two groups resided there. Bone stiffness was calculated using FEA and statistically correlated with the three-dimensional values of bone volume/tissue volume, bone mineral density, fractal dimension, trabecular separation, and trabecular bone pattern factor. Our method simplified the process for the assessment of trabecular bone stiffness by FEA from Micro-CT images and highlighted the importance of bone microarchitecture in conferring significantly increased bone quality capable of resisting failure due to increased mechanical loading.

## Introduction

Micro-computed tomography (Micro-CT) is a common tool in most bone biology laboratories and acknowledged as a valuable technique for investigating the microarchitecture of bone, for both *in vivo* and *ex vivo* applications (1–4). The Micro-CT derived images from reconstructed data accurately represent bone microstructural parameters for quantitative assessments (5). In addition, as the X-ray passes through the sample, the relative linear attenuation of the sample is represented on the image projections as values of gray scale (6). Corresponding X-ray attenuation on reconstructed images can thus be converted to bone mineral density (BMD) by calibrating the values with a tissue “phantom” of known mineral density. For trabecular bone, the structural parameters derived from Micro-CT data are based on traditional static bone histomorphometry, which evaluated the thickness, connectivity, distribution, and spacing of the trabecule. It is important to acknowledge that image segmentation methods can also influence the correlation of structural parameters to the stiffness of the trabecular bone structure being modeled, as described in several key finite element analysis (FEA) publications (7–9). Our key message remains in the added utility of initial selection of an appropriate region of bone for analysis (informed by grayscale attenuation) for building that FEA model. Measured parameters by Micro-CT including bone volume/tissue volume (BV/TV; %), trabecular number (Tb.N; mm^-1^), trabecular thickness (Tb.Th; mm), and trabecular separation (Tb.Sp; mm) have all been successfully utilized to assess the microarchitecture of rodent trabecular bones (5). In addition, there are other parameters that can also be used to represent bone microarchitectural complexity, such as fractal dimension (FD), trabecular bone pattern factor (Tb.Pf; 1/mm), degree of anisotropy (DA), and connectivity density (Conn.Dn; 1/mm^3^) (10). Although those parameters are well established with their own definitions, they remain indirect indices and require other computational tools to interpret bone stiffness and potential for mechanical failure (i.e., fracture) (11, 12). Resultantly, in bone research, the numerical method, finite element, was applied to assist in analyzing bone stiffness (13, 14).

Finite element method is founded on numerical methods for calculating the mechanics in various fields in industries. For bone analysis, the procedure starts by acquiring computed tomography images to build the geometry of the bone, which may include both trabecular and cortical components. Each voxel in the image stacks can be converted to an element in the finite element mesh. The material properties required for the analysis can also be inferred from the gray scale values on the images to enable a more accurate representation of the local stiffness of different bone structures (15–17). This process has been successfully implemented in many animal models (18), including modeling the entire rat vertebra, the vertebral body (without the top and bottom growth plates), and the trabecular bone compartment alone (1, 19). Nevertheless, the procedure from image segmentation to finite element model can prove challenging to the non-engineer, and inappropriate bone modeling techniques may result in redundant calculation in FEA. Thus, our aim was to simplify the selection of Micro-CT derived bone geometry for application to FEA and subsequently correlate the FEA results to bone microstructural and density based material parameters as an initial rudimentary assessment of bone stiffness (20). Although previous investigators have plotted the 2D curves of bone structural parameters (21), we show in this manuscript the additional utility of using the curve of 2D structural parameters of bone from Micro-CT image data to inform the selection of bone for subsequent FEA. In addition, using this approach, regional trabecular structural parameters become directly correlated to regional trabecular stiffness from FEA, further informing the impact of the given experimental intervention. In addition, using this approach, regional trabecular structural parameters become directly correlated to regional trabecular stiffness from FEA, further informing the impact of the given experimental intervention.

Our study hypothesis was that structural and density based parameters of trabecular bone microarchitecture from Micro-CT can be correlated to bone stiffness.

## Methods

### Animals

About 20, 3-month-old female, Sprague-Dawley rats were obtained from Charles River. The protocol pertaining to all procedures and aspects of the study was approved by the University of Alberta animal care and ethics committee. Before arrival, to induce an osteoporosis (OP)-like increase in bone resorption, ten rats were ovariectomized (OP-OVX); the remaining ten rats were sham-operated as normal controls (OP-Sham). All rats were euthanized 12 weeks after OVX and/or sham surgery and lumbar vertebrae 4–6 (L4–L6) were dissected. The muscle and soft tissues connected to vertebrae were removed, and samples were wrapped in paper towels dampened with phosphate buffered saline and stored frozen at -30°C for subsequent Micro-CT scan.

### Micro-CT imaging

**Figure 1:**
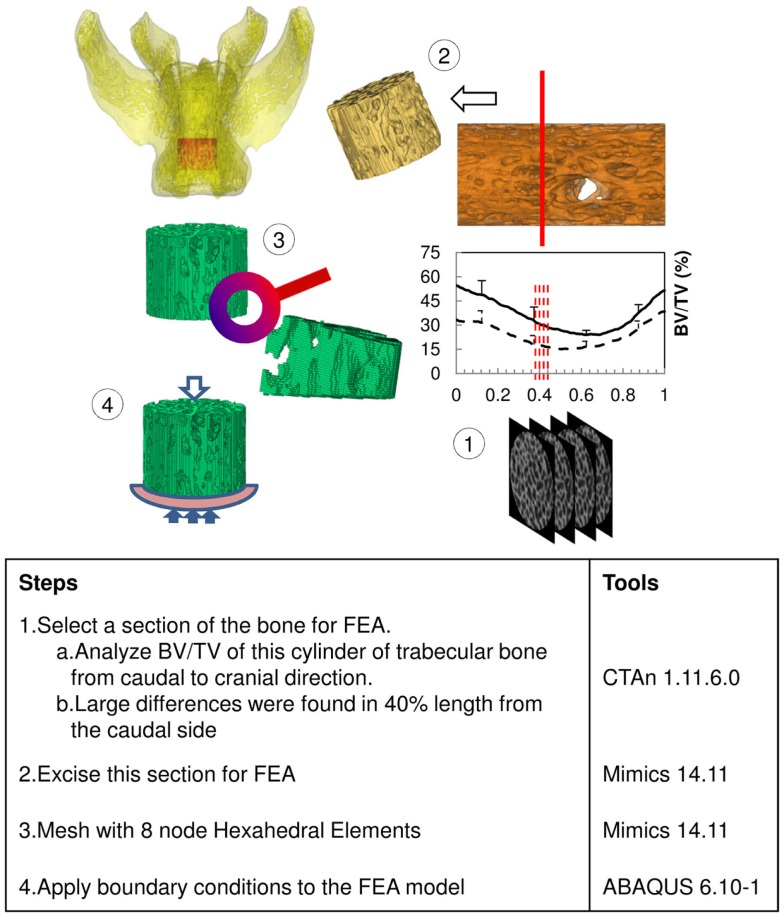
**Steps for calculating stiffness of trabecular bone cylinder of rat vertebra**.

The excised vertebrae, L4, L5, and L6, were thawed at room temperature for 2 h before scanning. All vertebrae were imaged using a benchtop Micro-CT imager (SkyScan 1076; Bruker-MicroCT, Kontich, Belgium) at 18 μm voxel image resolution with 70 kV, 100 μA, and a 1.0 mm aluminum filter. Projection images were reconstructed using bundled vendor software (Nrecon 1.6.1.5; Bruker-MicroCT, Kontich, Belgium). Dataviewer 1.4.3 software was employed to orient the cross sectional images parallel to the transaxial plane. A cylindrical region of interest (ROI) was segmented from trabecular bone within the vertebral body. The diameter of each cylinder was constrained by the endocortical bone margin enclosing the vertebra. The vertebral growth plates at each end were used to landmark the top and bottom segmentation boundaries. We utilized CTAn 1.11.6.0 (Bruker-MicroCT, Kontich, Belgium) to morphometrically analyze the trabecular bone cylinder. In order to calibrate BMD with Hounsfield units (HU), two hydroxyapatite [Ca_10_(PO_4_)_6_(OH)_2_] phantoms (Computerized Imaging Reference Systems Inc., Norfolk, VA, USA) with BMD 0.250 and 0.750 g/cm^3^ were included in the calculation based on the assumption that HU_air_ = -1000 and HU_water_ = 0. The threshold, 70 ~ 255, was chosen to segment out bone on 8-bit (0 ~ 255 gray level) bitmap (BMP) images. The measured structural parameters were BV/TV, BMD, Tb.Th, Tb.Sp, FD, Tb.Pf, DA, and Conn.Dn. Those parameters were calculated three dimensionally (3D) based on the volume of the cylinder. Except for Conn.Dn, all structure parameters were also calculated two dimensionally (2D) from cross sectional images slice by slice, along the longitudinal direction from caudal to cranial (Figure 1), and graphed with respect to the normalized height of the cylinder. The results were compared between groups and different levels of vertebrae in the same group.

### Finite element analysis

**Figure 2:**
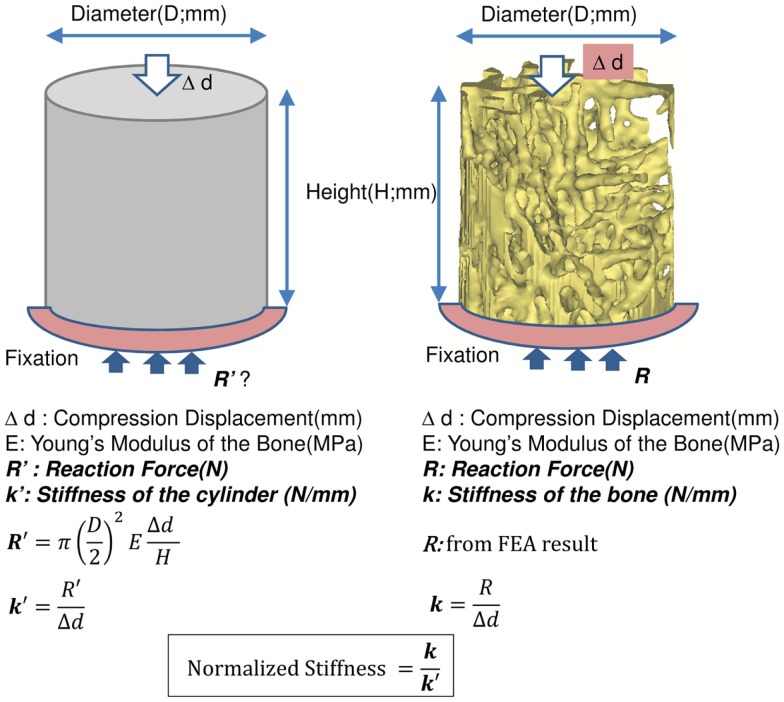
**Calculations of the index of stiffness (k/k′)**.

The results of the Micro-CT structural parameters indicated that the largest difference between groups were in the 40% portion of the cylindrical length from the caudal side. Thus, it was selected for FEA (Figure 1). The cross sectional images of this region were imported into Mimics 14.11 (Materialise, Leuven, Belgium) to build finite element meshes. Since the voxel of the Micro-CT data was 18 μm (≈17.156 μm), that dimension was used to build voxel mesh (hexahedral elements) for FEA to maintain the details of the microarchitecture. To avoid losing or distorting microarchitecture of trabecule, no resizing or smoothing was undertaken prior to meshing. After creating hexahedral elements (hex8), the meshes were exported as an input file (*.inp) format of ABAQUS (SIMULIA, Providence, RI, USA). The remaining pre-processing to include material properties and boundary conditions were done in ABAQUS/CAE 6.10-1. Since bone is expected to behave elastically and failure expected with small strain (22), the analysis was set as linear and elastic with Young’s modulus (E) = 24.5 GPa and Poisson’s ratio (ν) = 0.3 (23). All vertebrae were assigned with the same material property, as no significant difference of material properties was measured between normal and OVX bone (23). The bottom of the cylinder was fixed and a displacement (Δd), 5% of the height of the cylinder (strain = 0.05), was applied to the top of the cylinder. The stiffness (k) of the bone was calculated by dividing the summation of the vertical reaction forces (R) on the fixation to the applied displacement. Since the sizes of individual vertebrae varied, the stiffness (which comprises information of geometry and material property) would also vary with size. Therefore, values of k were further normalized with the theoretical stiffness (k′), which was calculated from the size of each cylinder by assuming that the cylinder was solid (Figure 2). The index of bone stiffness was represented as k/k′.

### Statistics

PASW^®^ Statistics 17.0 was used for statistical evaluations. The two-independent-samples test, Mann–Whitney *U* test, was used to find differences between individual groups on the same level of vertebra. This method was applied to both the results of Micro-CT structure parameters and the stiffness calculated by the FEA. The difference among three levels (L4, 5, and 6) of the same group was tested by Kruskal–Wallis *H* test. The Mann–Whitney *U* test was done to understand the difference between two individual vertebral levels. Results were shown as mean ± SD. An asymptotic value (*p*-value) <0.05 was used to evaluate the significance of differences. The correlation between Micro-CT structural parameters and the index of stiffness was described with Pearson correlation coefficients (*r*). The significance level was set at 0.05 (Sig. <0.05) with the two tailed test.

## Results

### 3D structural parameters

For the same level (i.e., Micro-CT slice) of vertebrae, most computed indices showed significant differences between OP-Sham and OP-OVX, except Tb.Th and DA. No significant difference of Tb.Th was found between OP-Sham and OP-OVX in the vertebrae of this OP-like rat model; however, the formation of new trabecular bone at the vertebral growth plates in these juvenile (3-month-old) rats proceeded as for normal (OP-Sham) rats. Since OP is a systemic metabolic disease, all vertebrae should experience the same changes after OVX. Therefore, in addition to comparing the results of each vertebral body at the same level, the morphometric changes from L4 to L6 in the same group were analyzed to provide further evaluation of OP-like bone change.

The different patterns of structural parameters among the same group and between the two groups (i.e., OVX-Sham vs. OVX-OP) were investigated to evaluate the spatial effect of OP in successive lumbar vertebral body segments (i.e., L4–L6). When comparing values between different levels of vertebrae in OP-Sham, no obvious trends between L4 and L6 were observed. However, in both groups, BMD, Tb.Th, and DA showed significant differences between L4 and L6 (L6 > L4), as well as L5 and L6 (L6 > L5). FD in OP-OVX showed significant differences among vertebral levels (Table 1) with the values increasing with sequential vertebral segment. This phenomenon was not seen in OP-Sham.

**Table 1:** **3D structure parameters of rat lumbar vertebrae calculated from Micro-CT images**.

		Treatment							
		OP-Sham				OP-OVX			
		L4	L5	L6	Total	L4	L5	L6	Total
		(*n* = 10)	(*n* = 10)	(*n* = 10)	(*n* = 30)	(*n* = 10)	(*n* = 10)	(*n* = 10)	(*n* = 30)
BV/TV (%)	Mean	34.3	32.8	39.0	35.4	20.0^c^	23.3^c^	26.7^a^^,^^b^	23.3
	SD	5.4	7.5	5.3	6.5	3.8	3.3	3.1	4.3
BMD (g/cm^3^)	Mean	0.207^c^	0.251^c^	0.304^a^^,^^b^	0.254	0.066^b^^,^^c^	0.157^a^^,^^c^	0.239^a^^,^^b^	0.154
	SD	0.035	0.069	0.075	0.072	0.024	0.062	0.060	0.088
Tb.Th (mm)	Mean	0.104^c^	0.104^c^	0.114^a^^,^^b^	0.108	0.106^c^	0.109^c^	0.114^a^^,^^b^	0.110
	SD	0.006	0.010	0.006	0.009	0.004	0.004	0.004	0.005
Tb.Sp (mm)	Mean	0.242^c^	0.251	0.264^a^	0.252	0.410	0.384	0.367	0.387
	SD	0.015	0.019	0.025	0.022	0.078	0.063	0.047	0.064
FD	Mean	2.40	2.38	2.43	2.40	2.23^b^^,^^c^	2.27^a^^,^^c^	2.31^a^^,^^b^	2.27
	SD	0.05	0.10	0.04	0.07	0.05	0.03	0.03	0.05
Tb.Pf (1/mm)	Mean	−1.53	−0.54	−3.54	−1.87	5.46^c^	4.02^c^	2.60^a^^,^^b^	4.03
	SD	2.84	4.85	2.57	3.67	1.81	1.16	1.34	1.85
DA	Mean	0.806^c^	0.777^c^	0.585^a^^,^^b^	0.723	0.901^b^^,^^c^	0.815^a^^,^^c^	0.702^a^^,^^b^	0.806
	SD	0.099	0.103	0.135	0.148	0.061	0.101	0.088	0.117
Conn.Dn (1/mm^3^)	Mean	84.6^c^	87.1^c^	69.0^a^^,^^b^	80.2	38.5	40.6	45.4	41.5
	SD	15.0	17.8	7.3	15.9	8.3	5.4	6.2	7.1

*Data expressed as mean and SD*.

**p*–value <0.05 means significantly different from other; *p*–value >0.05 was shaded with gray comparing to the other treatment*.

*^a^Significantly different from L4 under the same treatment*.

*^b^Significantly different from L5 under the same treatment*.

*^c^Significantly different from L6 under the same treatment*.

*BV/TV, bone volume/tissue volume; BMD, bone mineral density; Tb.Th, trabecular thickness; Tb.Sp, trabecular separation; FD, fractal dimension; Tb.Pf, trabecular bone pattern factor; DA, degree of anisotropy; Conn.Dn, connectivity density*.

### 2D structural parameters

**Figure 3:**
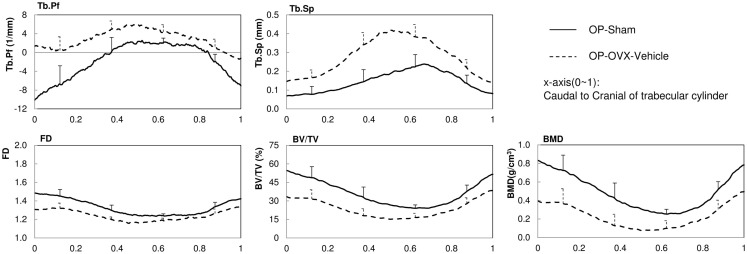
**2D structural parameters, BV/TV(%), Tb.Pf(1/mm), FD, Tb.Sp(mm), BMD(g/cm^3^), of the cylinder trabecular bone along the longitudinal axis from the caudal to cranial direction**. x-axis was normalized by the height of each vertebral body (trabecular cylinder) (x-axis: Caudal to Cranial; 0–1).

**Figure 4:**
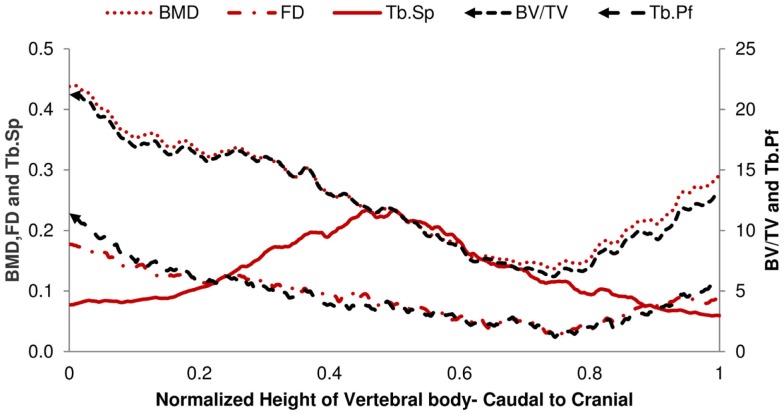
**Difference of 2D Micro-CT structure parameters between OP-Sham and OP-OVX-Vehicle**. The maximum difference of Tb.Sp is in the middle of the vertebra. All other curves show the minimum difference in the middle right of the vertebra body.

The structural parameters of image slices were calculated and plotted as curves to show the variations along the caudal-cranial direction. As the curves in the same group showed the same shapes, the curves of L4, L5, and L6 were averaged to represent each group. The curves of BV/TV, BMD, and FD were measured to decrease toward the middle; the curves of Tb.Sp and Tb.Pf increased toward the middle (Figure 3). In accordance with the observation from 3D models built from Micro-CT images, this reflected the vascular foramina present in the vertebral body. Regarding the subtractions between groups, only Tb.Sp showed the opposite phenomena, since its measurement was magnified by the anatomical structure, namely the vascular foramen (Figure 4).

### Finite element analysis

Based on the reaction force extracted from FEA, the normalized stiffness values (k/k′) were 0.383 ± 0.092 for OP-Sham and 0.141 ± 0.053 for OP-OVX. This stiffness index of OP-OVX was significantly less (*p* < 0.05) than that for OP-Sham. The Micro-CT structural parameters of this 40% length of the trabecular cylinder were also calculated in 3D. The results showed that Tb.Th. was not different (*p* > 0.05) between OP-Sham and OP-OVX. The Pearson’s correlation coefficients showed that BV/TV, BMD, and FD were positively correlated with the index of stiffness, and the correlation was significant at the 0.05 level. On the contrary, Tb.Sp and Tb.Pf were negatively correlated with the index of stiffness. The values of Conn.Dn were significantly different between groups; yet, they showed no significant correlation to the calculated stiffness (Table 2).

**Table 2:** **3D structural parameters and index of stiffness of the 4/10 length L4**.

			Treatment			
			OP-Sham		OP-OVX	
			L4 (*n* = 10)		L4 (*n* = 10)	
BV/TV (%)	Mean	*r*	41.7	0.934	23.1	0.965
	SD	Sig.	7.2	0.000	5.2	0.000
BMD (g/cm^3^)	Mean	*r*	0.248	0.674	0.083	0.954
	SD	Sig.	0.032	0.032	0.030	0.000
Tb.Th.(mm)	Mean	*r*	0.102	0.817	0.103	0.726
	SD	Sig.	0.006	0.004	0.005	0.018
Tb.Sp.(mm)	Mean	*r*	0.166	-0.838	0.341	−0.784
	SD	Sig.	0.013	0.002	0.166	0.007
FD	Mean	*r*	2.48	0.928	2.26	0.948
	SD	Sig.	0.06	0.000	0.06	0.000
Tb.Pf (1/mm)	Mean	*r*	−4.00	−0.956	5.46	-0.965
	SD	Sig.	4.08	0.000	2.48	0.000
DA	Mean	*r*	0.557	0.238	0.525	0.502
	SD	Sig.	0.044	0.508	0.056	0.139
Conn.Dn (1/mm^3^)	Mean	*r*	131.6	0.542	55.2	0.648
	SD	Sig.	23.8	0.105	13.7	0.043
k/k′	Mean	*r*	0.383	1	0.141	1
	SD	Sig.	0.092		0.053	

*Data expressed as mean and SD*.

**p*–value <0.05 means significantly different from the other group; *p*–value >0.05 was shaded with gray*.

*The significant level of Pearson’s correlation coefficient (*r*) was set at 0.05 and means no significant correlation to k/k′; the value (Sig.) >0.05 was shaded with light up diagonal*.

*BV/TV, bone volume/tissue volume; BMD, bone mineral density; Tb.Th, trabecular thickness; Tb.Sp, trabecular separation; FD, fractal dimension; Tb.Pf, trabecular bone pattern factor; DA, degree of anisotropy; Conn.Dn, connectivity density; k/k′, normalized stiffness value*.

## Discussion

Our study details an alternative approach for FEA of bone, whereby Micro-CT derived grayscale attenuation and segmentation data serve initially to guide the selection of trabecular bone regions for analysis by FEA. Thus, instead of modeling the entire bone with FEA by default (currently the most common approach employed), our method will determine sample regions with “distinct differences” in the trabecular structure (informed by grayscale attenuation and segmentation data) that will be modeled to subsequently identify the relationship between trabecular structure and stiffness. Future implementation of this approach would greatly simplify the assessment of trabecular bone structural parameters and the distribution of stiffness in regional bone locations (e.g., for high-throughput preclinical applications, such as gaging the antiresorptive efficacy of a novel OP drug), without the need for exhaustive computational modeling of the entire bone structure.

This study recapitulates the importance of trabecular bone microarchitecture in conferring structural strength to otherwise similar trabecular bone geometry. In this study, hormonal depletion induced in female rats (secondary to ovariectomy surgery) served to imbalance the bone remodeling process toward systemic bone resorption and decreased bone volume. Despite that systemic bias, no significant difference of Tb.Th between OP-Sham and OP-OVX groups were measured in vertebral trabecular bone using Micro-CT, suggesting that microarchitectural differences were responsible for the significantly reduced structural strength measured in OP-OVX rats.

### Resolution issues of images in micro-CT

The selection of resolution for Micro-CT imaging is a critical parameter that, if not chosen adequately, would have high impact on the analysis results. The average trabecular thickness of OP-Sham obtained from the analyzed images was 108 ± 9 μm (Table 1). That number was obtained from images scanned with a target resolution of 18 μm. In theory, other resolutions (9 and 35 μm) available through the Micro-CT imager in our laboratory are feasible for scanning rat vertebra. However, the 18 μm resolution meant that each trabecula would contain six voxels along the thickness direction, which is adequate to capture the complex nature of the trabecular structure. In addition, the finite element mesh with six elements per trabecular thickness is considered adequate for the model accuracy. It is worth noting that a possible advantage associated with scanning with a higher resolution is to observe possible microcracks in the trabecular bone. Nevertheless, scanning our samples with a higher resolution (9 μm) was not practical because of the cumbersome file size of the resulting image and the associated doubling of scanning time.

### Threshold selection for segmenting bone in image processing

Besides the resolution, the threshold for segmenting bone is also essential, since the results of the variation along the trabecular longitudinal axis may change with different thresholds. Thus, consistent threshold should be applied in the same study to exclude differences resulting from variations in thresholds. In general, for calculating the BMD of trabecular bone among different groups, if the HU differences were not significant between groups, the trend of BMD variations should be very similar to those of BV/TV in the 2D curves (Figure 3). This was observed from our results and served as evidence of no significant HU variations between normal and OP bone.

### Bone selection for FEA and the advantage of our model

In rodents, histomorphometric measurements of bone remodeling are complicated by concurrent longitudinal bone growth, which expands both the bone diameter as well as trabecular bone at the growth plates. The bone remodeling, which was influenced by ovariectomy, would best be evidenced on trabecular surfaces. Our analysis of segmented trabecular cylinders excluded bone near the growth plates as well as cortical bone. Excluding cortical bone in FEA to evaluate stiffness allowed us to isolate the effect of OP on the stiffness of the trabecular bone. Furthermore, in our model, we analyzed 40% of the trabecular cylinder starting from the caudal side. The values of DA were also reduced by selecting only 40% of the trabecular cylinder. It means the structure changed from being anisotropic toward isotropy (Tables 1 and 2) (24, 25). The advantage of using only this selection is in avoiding the foramina effects which may reduce the difference between groups. Furthermore, the model with 40% of the length has fewer elements than the model of entire vertebra or trabecular bone. This led to less computational time for the FEA.

### Material properties of finite element model

A limitation to our study was ignoring the mapping of material properties according to different HU or gray values. The Young’s modulus in each trabecula can be correlated to values of HU (26). However, several studies on rat bone of OVX (0.91 ± 0.13 and 21.01 ± 2.48 GPa) and sham (0.90 ± 0.09 and 22.03 ± 2.44 GPa) groups showed no significant difference in the hardness and elastic modulus (23, 27). Hence, the same material properties were assigned for the two groups in this study. If further mechanical analysis was required, such as permanent deformation, viscosity, or time effect and crack propagation, then material properties would need to be assigned specifically and non-linear FEA could be applied.

## Conclusion

In this paper, we presented a simple process by which the stiffness of trabecular bone can be inferred from structural parameters, without the need for computationally exhaustive FEA models. The 2D structural parameters calculated from Micro-CT analysis reflected the difference in the geometry of the bone between the sham and the OP group. The changes in the following Micro-CT parameters (BV/TV, BMD, Tb.Sp, FD, Tb.Pf, and Conn.Dn) between the two groups were examined and compared with the stiffness obtained from FEA. Other than for Conn.Dn, the values of BV/TV, BMD, FD, Tb.Sp, and Tb.Pf were shown to be related to the structural stiffness. This approach can be readily applied to assess trabecular bone structural changes to quickly infer stiffness, particularly during longitudinal “*in vivo*” studies of laboratory rodents, or to assess the effects of drug treatments.

## Conflict of Interest Statement

The authors declare that the research was conducted in the absence of any commercial or financial relationships that could be construed as a potential conflict of interest.
